# Long-term Intracellular Recording of Optogenetically-induced Electrical Activities using Vertical Nanowire Multi Electrode Array

**DOI:** 10.1038/s41598-020-61325-3

**Published:** 2020-03-09

**Authors:** Jisoo Yoo, Hankyul Kwak, Juyoung Kwon, Go Eun Ha, Elliot H. Lee, Seungwoo Song, Jukwan Na, Hyo-Jung Lee, Jaejun Lee, Areum Hwangbo, Eunkyung Cha, Youngcheol Chae, Eunji Cheong, Heon-Jin Choi

**Affiliations:** 1 Department of Materials Science and Engineering, Yonsei University, Seoul, 03722 Republic of Korea; 2 Department of Biotechnology, College of Life Science and Biotechnology, Yonsei University, Seoul, 03722 Republic of Korea; 3 Department of Electrical and Electronic Engineering, Yonsei University, Seoul, 03722 Republic of Korea; 4 Center for Nanomedicine, Institute for Basic Science (IBS), Seoul, 03722 Republic of Korea

**Keywords:** Biological techniques, Biophysical methods, Electrophysiology, Nanoscience and technology, Nanobiotechnology

## Abstract

Continuous recording of intracellular activities in single cells is required for deciphering rare, dynamic and heterogeneous cell responses, which are missed by population or brief single-cell recording. Even if the field of intracellular recording is constantly proceeding, several technical challenges are still remained to conquer this important approach. Here, we demonstrate long-term intracellular recording by combining a vertical nanowire multi electrode array (VNMEA) with optogenetic stimulation to minimally disrupt cell survival and functions during intracellular access and measurement. We synthesized small-diameter and high-aspect-ratio silicon nanowires to spontaneously penetrate into single cells, and used light to modulate the cell’s responsiveness. The light-induced intra- and extracellular activities of individual optogenetically-modified cells were measured simultaneously, and each cell showed distinctly different measurement characteristics according to the cell-electrode configuration. Intracellular recordings were achieved continuously and reliably without signal interference and attenuation over 24 hours. The integration of two controllable techniques, vertically grown nanowire electrodes and optogenetics, expands the strategies for discovering the mechanisms for crucial physiological and dynamic processes in various types of cells.

## Introduction

Critical cellular dynamics, including transcriptional change, protein synthesis, receptor replacement, and synaptic plasticity in neural cells, take place over time periods ranging from several hours to days^1^. A common approach for discerning these cellular mechanisms in the single cell level is to measure its intracellular electrical activity using sharp microelectrodes or patch-clamping. However, as intracellular recording using these conventional electrodes is achieved by tearing the cell membrane, disruption of cell integrity limits the recording duration to several hours^2^, providing only brief ‘snapshots’ of cellular dynamics during limited experimental sessions^3^. Thus, such intermittent recordings have limitations in tracking single-unit activity over timescales relevant for most developmental and learning processes, or for pharmaceutical drug screening over long periods.

Three-dimensional micro/nanostructure electrodes have shown superior feasibility for the electrophysiological study of single cells by accessing the cell interior and recent technologies allow simultaneous observations of the intracellular activity of individual cells in neuronal populations with high temporal/spatial resolution^4–17^. However, despite their advantages of high-sensitivity and minimal invasiveness, the reported time durations of intracellular recording using micro/nanostructure-based electrodes have not exceeded 80 min^4^. These intracellular recordings have been demonstrated by employing one of two agents: (i) external poration force achieved by a burst of electrical (electroporation) or optical (optoporation)^4^ pulses applied through electrodes to increase the permeability of the cell membrane and promote electrode penetration, or (ii) depolarizing stimulus that raises the membrane potential above the action potential (AP) threshold to induce spiking activities^7,8,10,11^.

However, even if localized membrane poration has been shown to detect intracellular action potentials with a good signal-to-noise ratio by forming membrane pores, intracellular access by membrane poration is transient because of spontaneous membrane resealing^6^, and hence additional pulses are required to prolong the recording sessions. Previous measurements using metal (Au, Pt) nanopillars^5^, Iridium oxide (IrOx) nanotube^6^ and gold mushroom-shaped microelectrodes^9–11^ demonstrated that the cell membrane reseals in just a few minutes or a maximum of 1 h after electroporation, severely limiting the time duration of intracellular recording.

When using depolarizing stimuli for excitable cells to control polarization, pharmacological methods^12^ (such as using KCl, glutamate for excitation) and electric field delivered by bath solutions^9–11^ have been generally employed. Although these methods were easy and allowed temporal control, they were unable to provide site-specific localized control of target cells^13,14^. To address these limitations, a ‘patch-nanowire configuration’ was developed which allowed whole-cell patch-clamp recording or stimulation of cells residing on top of nanowire electrodes^7,9–11^. However, whole-cell patch manipulation, which is achieved by tearing an opening in the cell membrane via pipette suction, exposes the cell cytoplasm to the liquid-filled pipette electrode, rapidly disrupting cell integrity and inducing homeostasis between the cell cytosol and pipette liquid volume^2^. Consequently, the cell deteriorates within minutes or hours, preventing long-term recording.

To eliminate these two issues which are key factors for prolonging the duration of intracellular recording, we designed a (i) CVD-grown vertical nanowire multi-electrode array (VNMEA) that can spontaneously probe the cell interior without the perforation process, and (ii) illuminated light-sensitive transmembrane ion channels^18,19^ expressed on the membrane of optogenetically modified cells. We demonstrate stable long-term recording of intracellular electrical activity in optogenetically modified cells for over 24 h without amplitude attenuation. While traditional electrical stimulation, which has no built-in feedback, directly injects an external current without a specific ionic identity and is merely superimposed on the ongoing membrane potential dynamics^20,21^, current mediated by light induced ion-channels instantly responds to changes in transmembrane voltage during AP, resulting in real-time negative feedback control^22,23^. As this approach circumvents the drawbacks of traditional stimulation methods and shows excellent spatiotemporal resolution, we were able to obtain high-quality intracellular and extracellular recordings of individual cells. Moreover, the generation of light-induced artifacts, one specific challenge raised by the combination of electrophysiological recordings with optical stimulation^24,25^, was mitigated by minimizing the exposed metal surface of nanoelectrodes and limiting the duration of the light pulse^25^. As this system which integrates two controllable techniques, the fabrication of nanowire devices using complementary metal oxide semiconductor (CMOS) processes and optogenetics, is customizable for various cell types, it can elucidate crucial cellular dynamics which can be verified over a long period from several hours to days.

## Results

### Fabrication of VNMEA for interfacing cells

**Figure 1:**
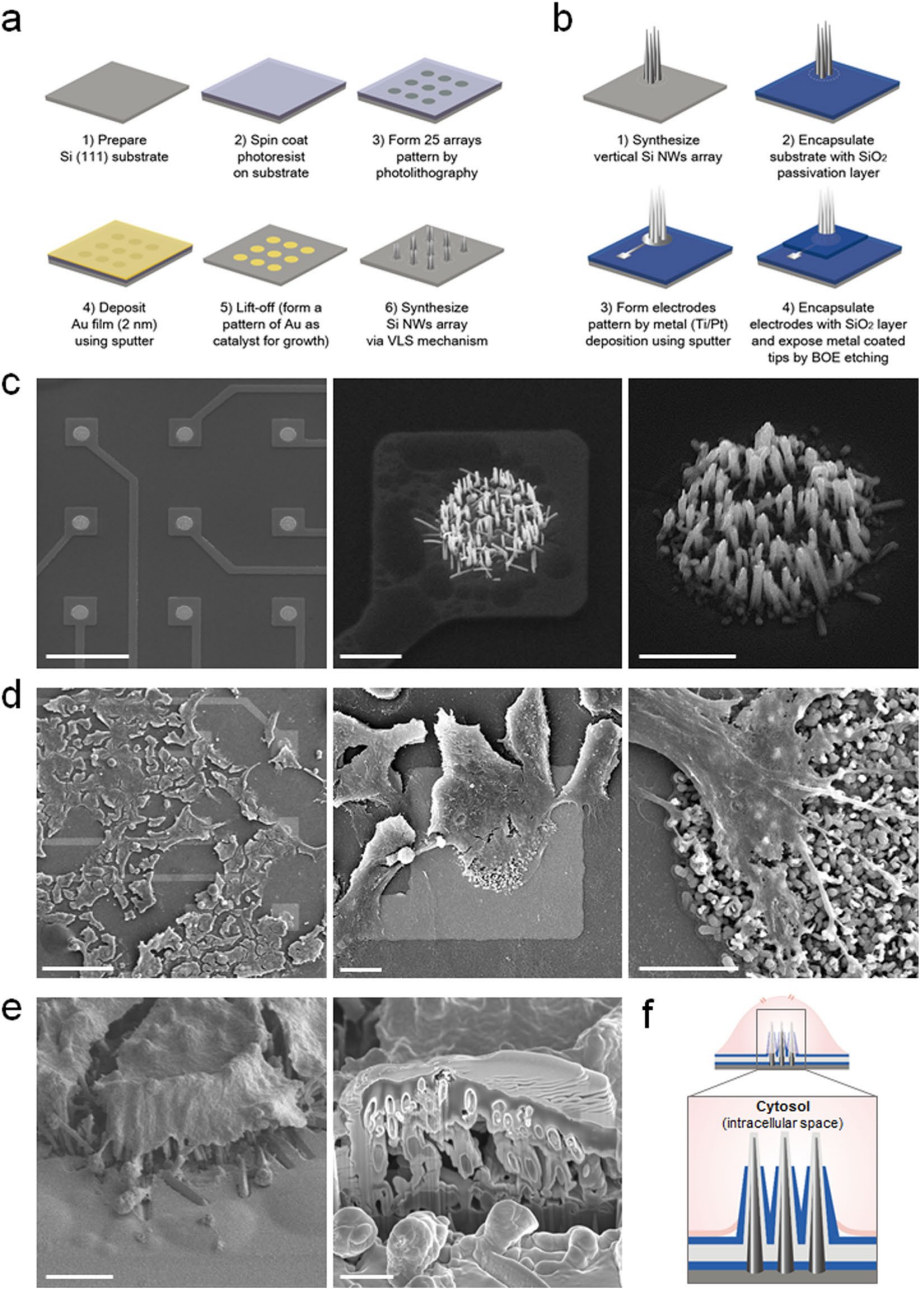
High-density VNMEA for intracellular access. (**a**) Schematic of the fabrication process for position-defined vertical Si nanowires arrays by lithographic method. (**b**) Experimental scheme of the VNMEA top-down fabrication procedure after synthesizing the patterned 25-arrays of vertical Si nanowires. (**c**) SEM images of site-specific nanowire electrodes which are grown by patterns of 20 μm diameter Au film discs. Scale bar, 100 μm, 5 μm, 1 μm. (**d**) SEM images of HEK293T cells cultured on VNMEA. Scale bar, 100 μm, 10 μm, 5 μm. (**e**) FIB cross section of HEK293T cells located on an electrode pad. Scale bar, 5 μm, 2 μm. (**f**) Schematic illustration of tight-seal conformation by high-density vertical nanowire electrodes accessing the cytosol.

Silicon (Si) nanowire substrates can be prepared by either growing the nanowires epitaxially from a Si wafer using chemical vapor deposition (CVD) or etching them from a wafer using standard semiconductor techniques. Nanowire electrodes produced for intracellular recording are generally fabricated by etching the extraneous materials from the wafer, due to the ease with which ordered arrays of nanowires can be constructed. This facilitates the electrical contact to the nanowires and their integration into large-scale devices. However, these nanowire electrodes fabricated by top-down methods cannot be used for spontaneous cell penetration without external forces^5,7–12^. In this study, we synthesized 25-arrays of vertically aligned Si nanowires on Si (111) substrates via the VLS (vapor-liquid-solid)-CVD method. As CVD-grown Si nanowires have high-aspect-ratios (<10^3^) and are sufficiently rigid to be mechanically manipulated, their nanometer scale diameters and high-aspect-ratios allow them to access the interiors of living cells without an external force for penetration. Previous studies have shown that CVD-grown vertical nanowires spontaneously penetrate the cell membrane when cells are placed on top of the nanowires^26–31^. The biocompatibility of small diameter nanowires is a critical element for the *in situ* study of cellular processes of living cells^26^. Thus, the density, diameter, and height of the nanowires are precisely controlled by varying the processing parameters so that their spontaneous intracellular access minimally disrupts cell survival and functions. To facilitate the electrical interface with individual cells, we synthesized arrays of vertical Si nanowires by the lithographic method for independent recording of single cell activity (Fig. 1a). By arranging the gold pads required for catalyzing the nanowires, we were able to position the nanowires with an optimum electrode pitch (110 µm) and dimensions for recording individual cells. For electrode fabrication, we used a top-down process with a CMOS device described in our previous reports^31,32^ (Fig. 1b). The nanowire electrodes are located at the center of each recording pad, and each nanowire is deposited with metal (titanium/platinum) for electrical coupling with the cell interior. They are encapsulated with SiO_2_ passivation layers of 300-nm thickness for close-sealing with the cell membrane, and Au-coated nanowire electrode tips were selectively exposed for intracellular recording (Fig. 1c).

The physical interaction between the nanowires and the cells were studied using scanning electron microscopy (SEM) and focused ion beam (FIB) milling (Fig. 1d,e). The high-density vertical nanowire arrays are able to support the growth of adherent cells and serve as better geometrical focal adhesion (FA) points for cell attachment than flat surfaces (Fig. 1d, Supplementary Fig. S1). The FA points increase their contact with nanowires by wrapping them with membrane. Moreover, for the characterization of the interface between the cellular membrane and nanowires, we performed sequential cross-sectioning of the cells using FIB and acquired images with SEM. In Fig. 1e, we could clearly observe the underlying nanowires penetrating the cell, forming close physical interaction, and generating effective seal formation which are crucial for reliably recording intracellular activities.

### Optogenetic modification of HEK293T cells to be optically controlled the cellular activity

**Figure 2:**
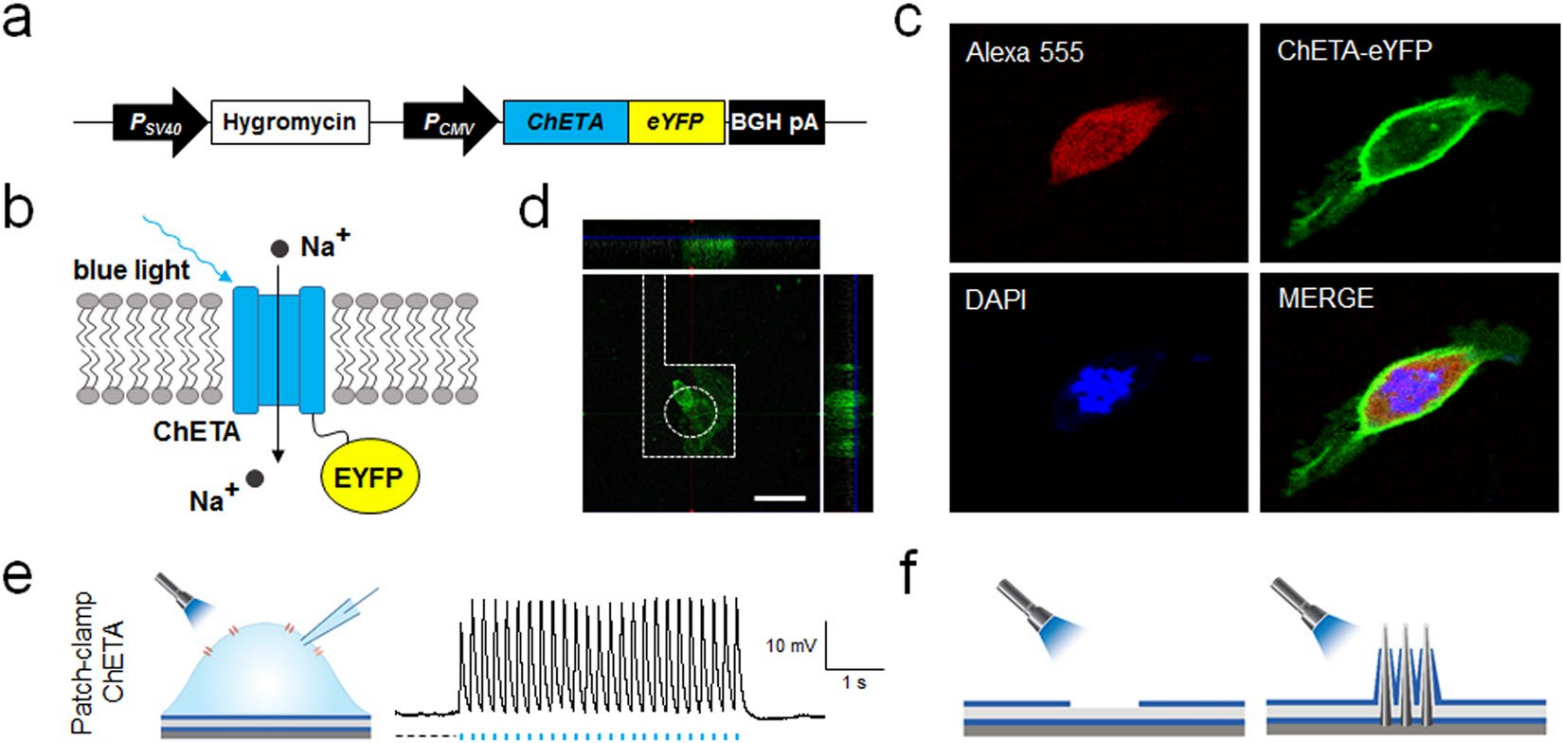
ChETA-mediated optogenetic modification of HEK293T cells and experimental approaches for minimizing optical interference. (**a**) Schematic illustration of the preparation of ChETA-expressing HEK293T cell. (**b**) Schematic illustration of membrane-inserted ChETA and enhanced yellow fluorescence protein (EYFP). Illumination of blue light induces the influx of cations via ChETA. (**c**) Confocal microscope images of ChETA-expressing HEK293T cells. (**d**) Confocal microscope image of ChETA-expressing HEK293T cells seated on a VNMEA pad. Scale bar, 20 μm. (**e**) Whole-cell patch clamp recording of ChETA-expressing HEK293T cells. (**f**) Schematics of solutions for mitigating the generation of light-induced artifact by minimizing the exposed metal (Pt) surface of electrodes.

As for second strategy to increase recording duration, we exploited optogenetic manipulation for activating cells instead of traditional excitation methods such as electric field or chemical agents. By artificially incorporating the light-sensitive protein to cell membrane, optogenetically modified cells show the light-induced ion channel response and also self-regulate their transmembrane voltage during generation of APs (real-time negative feedback control), minimizing the cell damage from over-responsiveness. To measure solely stimulus-evoked response without interference of spontaneous activity, we chose HEK293T cells for their lack of spontaneous electrical activities. We modified HEK293T cells to stably express light-activated cation channel, ChETA. ChETA, a variant of the channelrhodopsin (ChR2) channel, was selected for its faster kinetics which is suitable to accurately determine the response time of VNMEA^33^. When blue light is illuminated, ChETA is activated, and cations such as Na^+^ and Ca^2+^ ion flow in from the exterior to the interior of the cell via ChETA, inducing membrane depolarization. Figure 2a shows the modified gene set for ChETA expression. For visualization of ChETA expression, enhanced yellow fluorescence protein (eYFP) was co-expressed with ChETA. Figure 2b shows a schematic illustration of membrane-inserted ChETA-eYFP. Figure 2c shows confocal images of a HEK293T cell expressing ChETA-eYFP. For cytosol identification, Alexa 555 hydrazide (stained red) was infused after membrane permeabilization by Tween 20, and DAPI staining (stained blue) was also performed for nuclear identification. Note that eYFP (stained green) is highly expressed on the cell membrane, indicating the successful expression of ChETA on the membrane. Figure 2d shows z-axis view of confocal images of ChETA-expressing HEK293T cells on the VNMEA after one day *in vitro* (1-DIV). To physiologically confirm optogenetic modification of cells, light-evoked cellular activity was recorded by whole-cell patch clamp (Fig. 2e). ChETA potential was on-time recorded according to illumination of blue light (5 Hz with a pulse width of 20 ms).

### Experimental approaches for minimizing optical interference

The use of optical stimulation for activating cellular activities can induce various electrical artifacts during electrophysiological recordings. These artifacts, possible photoelectric effects, can be produced by laser beam striking the tip of a metal electrode, and are proportional in size to the total laser power and the amount of exposed metal. Thus, the primary strategy for minimizing optical interference is to limit metal exposure to light by limiting the duration of the light pulse and minimizing the total exposed metal surface. Though the electrode tip of VNMEA is made of coated Pt film, we minimized the exposed metal tip by designed selective tip-etching process and thus could mitigated the optical artifacts (Fig. 2f, Supplementary Fig. S2). The SiO_2_ passivation layer of the nanowire electrode tip could be selectively etched out by masking the bottom surface using poly (methyl methacrylate), exposing metal of the nanowire surface by using a buffered oxide etchant (BOE). For verifying the light-induced artifacts of VNMEA, we measured the electrical potential according to illumination of light pulses (same experimental protocol for electrophysiological recordings that are limiting the duration of pulse 20 ms), and compared with those of planar type (Pt film with pad diameter 20 µm) electrodes. Compared with the artifacts of planar type electrodes which are higher (40~80 mV) as action potentials (60~90 mV), those of VNMEA are negligible (5~10 mV) for recording the cellular activities.

### Intracellular and extracellular recording by VNMEA

**Figure 3:**
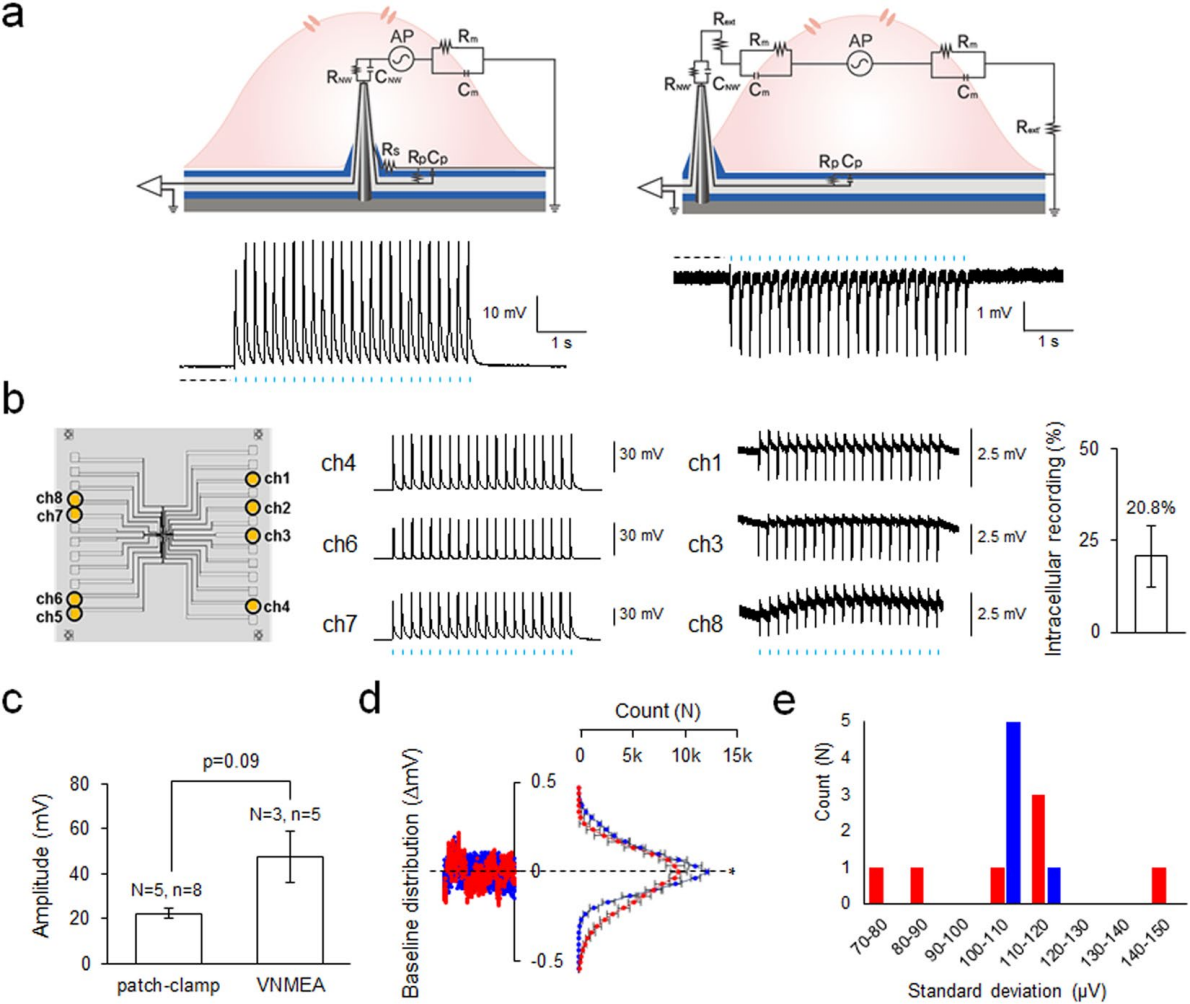
Intra/extracellular recording by VNMEA. (**a**) Equivalent circuit model and measured cellular potentials of intracellular (left) and extracellular (right) recording through the VNMEA. (**b**) Representative electrical potentials evoked by optic stimuli from each channel (left) and bar-graph of intracellular recording efficiency (right). (**c**) Bar-graph of amplitude of membrane potential evoked by blue-light illumination. Statistical significance was evaluated by Student’s t-test. (**d**) Count distribution analysis of baselines from intracellular recordings either by whole-cell patch clamp (red) or Si nanowires (blue). Note that Si nanowires (blue) show less variation of count, meaning stable baseline. Baselines of 1 s which were used in quantification are shown with dotted black lines in recording traces. (**e**) histogram of standard deviation of baseline potentials recorded by patch-clamp (red) or VNMEA (blue). Data are represented as mean ± SEM.

We investigated the correlations between cell-electrode configuration and the resulting recorded electrophysiological activity. We packaged our VNMEAs on customized printed circuit boards (PCB) and tested them using optogenetically-modified HEK293T cells activated by periodic optical pulses (Supporting Fig. S3). The amplitude and shape (phase) of recorded potentials are governed by the cell-electrode configuration, which can either be an intracellular or extracellular configuration (Fig. 3a). The electrochemical interface of the nanowire electrode in the intracellular or extracellular configuration form distinct equivalent circuits based on each component of the cell/electrode interface^7,31^. R_NW_ and C_NW_ represent the resistance and capacitance of the nanowire electrode in the intracellular environment. R_NW’_ and C_NW’_ represent the resistance and capacitance of the nanowire electrode in the environment of extracellular solution. R_m_ and C_m_ represent the characteristic resistance and capacitance of HEK293T cell. R_s_ represents the seal resistance formed between the nanowire electrode and cell membrane. R_p_ represents the leak resistance of electrode passivation and C_p_ represents parasitic capacitance of device. Intracellular and extracellular current flow produced by propagation of action potentials are expressed as alternating current power source AP. The equivalent circuit also includes extracellular solution resistance R_ext_ which represents resistance between the nanowire electrode and cell, and R_ext’_ which represents resistance between the cell and ground electrode. In accordance with the current flow generated by the potential difference between the depolarized and resting regions, intracellular potentials measured by electrodes accessing the cell interior show positive monophasic spikes with high amplitudes (range of 50–80 mV) resembling those from typical intracellular recordings using patch-clamps or microelectrodes. Whereas, measured extracellular potentials show negative biphasic spikes with low amplitudes (range of 2–3 mV) resembling those from other typical planar MEAs.

Our VNMEAs fabricated by lithographic methods can independently deliver the electrical potentials of single cells and can also perform parallel recordings, allowing the simultaneous measurement of multiple cells in a culture. Fig. 3b shows a recording by six different electrodes on a 5 × 5 array chip with 110 µm separation between adjacent electrodes. Three channels (Ch. 4, 6, and 7) measured intracellular signals featuring positive peak polarities with high amplitudes while the other three channels (Ch. 1, 3, and 8) measured extracellular signals featuring negative peak polarities with low amplitudes. As our VNMEA experimental setup does not require other external stimulation or special treatments such as electroporation, surface functionalization, or the addition of surfactants for intracellular interfacing, we could identify and distinguish the initial configuration of cells just by the result of recorded activities.

### Characterization of intracellular recording by VNMEA

To evaluate quality of intracellular recordings by VNMEA, we compared measurement results with the widely used whole-cell patch clamp intracellular technique (Fig. 3c–e). As the extracellular signal is an amalgamation of signals from multiple cells, we selectively quantified intracellular recordings from VNMEA for comparison with patch-clamp recordings. We found that ChETA-induced membrane potentials measured by VNMEA were marginally larger (p = 0.07) than those measured using a whole-cell patch clamp (Fig. 3c). This difference in peak amplitudes might originate from the cytosolic compositional change due to the whole-cell patch clamp. After rupture of the membrane during whole-cell patch clamping, the native cytosolic composition is replaced with the internal-pipette solution. That is, the signals are measured under artificial cytosolic composition, which will lead to changes in concentration gradients of charged ions between the exterior and interior of the cell. These differences in concentration gradients then lead to a change in cation influx by ChETA activation, and thereby changing the membrane potential. However, as VNMEA does not involve artificial internal-pipette solution, VNMEA can record cell signals from the original cytosolic composition. This systemic difference between the patch-clamp and VNMEA may have caused the difference in observed peak amplitudes.

Most reported MEAs thus far can only detect extracellular signals from cells because of their planarity^34^. Thus, they have lower SNR compared to intracellular recording techniques such as the whole-cell patch clamp. In addition, low-amplitude events (<10 pA) such as miniature excitatory postsynaptic currents (mEPSCs) cannot be detected extracellularly. The nonlinear signal distortion during transduction at cell-electrode interfaces is still an issue for MEAs due to poor sealing resistance and high levels of current leakage. To overcome these limitations, vertical nanowires have emerged as a novel technique for intracellular recording, with some studies successfully able to detect intracellular signals. However, signal attenuation and unstable baselines were raised as drawbacks of nanowire systems.

Baseline stability is another important factor for electrophysiological recording devices. To evaluate the stability of the baseline, we analyzed the distribution of baseline potentials (Fig. 3d). First, the deviation of each acquired potential value from the baseline potential in a 1 sec time frame was calculated and the count of deviations was averaged and plotted (red, patch-clamp; blue, VNMEA). The analyzed sections are marked with black dotted lines in the traces. A higher count at baseline potential signifies a more stable baseline. The count value near 0 in VNMEA was significantly higher than that of the patch-clamp, indicating a more stable baseline for VNMEA. Figure 3e shows the histogram of standard deviations (sigma, σ) of baseline potentials recorded by patch-clamp (red) and VNMEA (blue). The values of σ with the patch-clamp showed a broad range between 70 and 150 µA. Meanwhile, the values of σ with VNMEA showed a relatively narrow range between 100 and 120 µA. This indicates that baseline signals recorded by VNMEA have a higher uniformity than those from the patch-clamp. Our results clearly demonstrate the unique characteristics of VNMEAs for high-precision and stable recording of intracellular signals.

### 24 h intracellular recording by VNMEA

**Figure 4:**
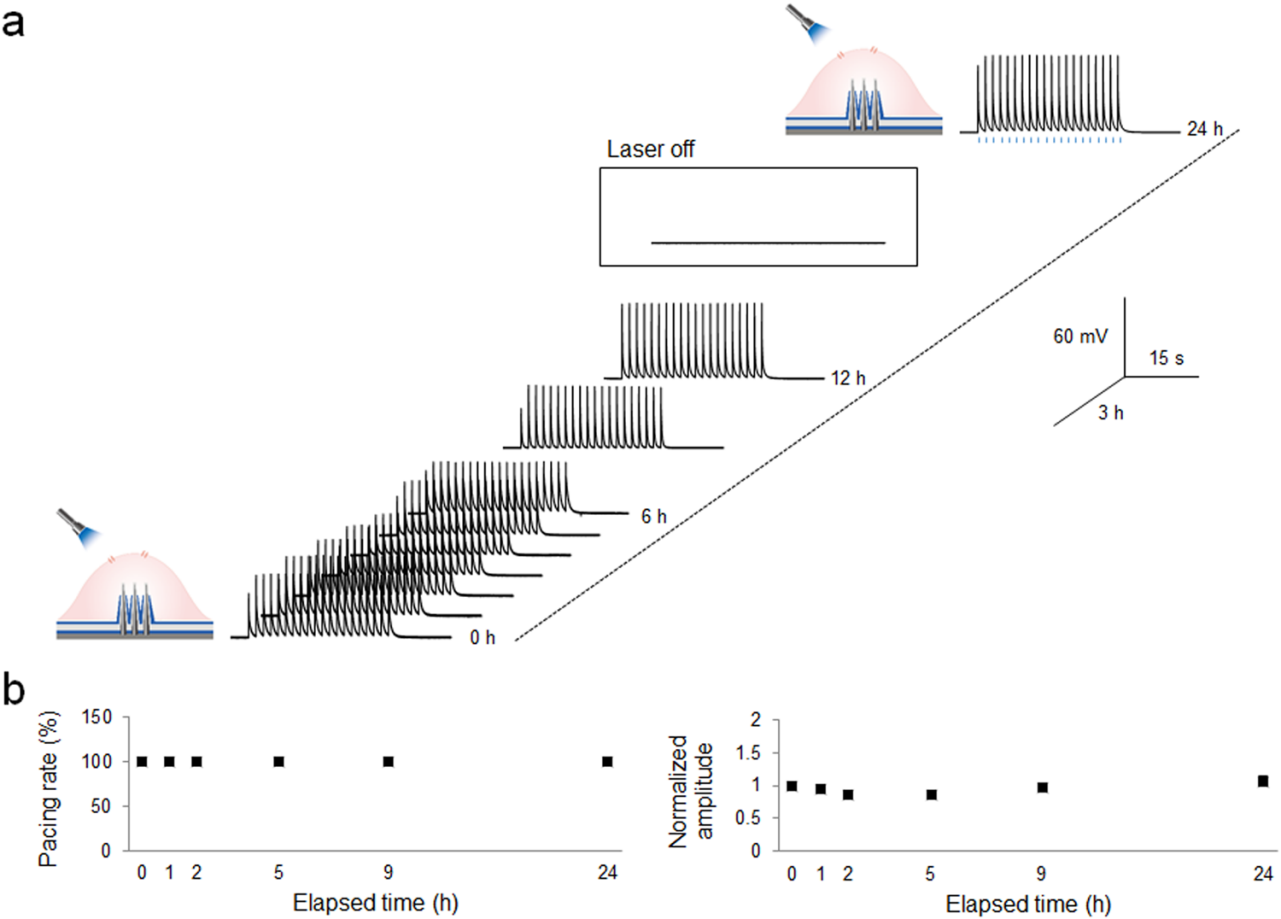
Long-term intracellular recording of ChETA-expressing HEK293T cells by VNMEA. (**a**) Traces of potential changes by ChETA activation acquired from VNMEA for 24 h with periodic optic stimulation. (**b**) Success rate of pacing by optic stimulation and normalized amplitudes. Note that within 24 h, both pacing rate and normalized amplitude showed no significant differences. Signals were normalized to the amplitudes of fifth responses of 0 h recording.

To assess the superiority of VNMEA for long-term recording, we activated ChETA by optical stimulation and successfully measured the ChETA potentials for 24 h (Fig. 4). We measured the intracellular potentials of ChETA at specific times, and replaced the culture medium periodically. Amplitudes were quantified by the fifth peak of each measurement and normalized to the fifth peak of the 0 h measurement. The measurements revealed that optogenetic stimulation of ChETA successfully induced on-time depolarization with a 100% pacing rate. Also, the normalized data for peak amplitude showed no significant differences among measured amplitudes at any time (maintained at 60 mV). These long-term physiological measurements of ChETA demonstrate the stable and compatible cell-coupling of VNMEA with optogenetic stimulation. The long-term recording efficiency of VNMEA might be derived from several factors: (1) By spontaneously accessing the cell interior with a bundle of high-density nanowires and maintaining the nanowires inside the cell, we could minimize the cellular impacts which can be aroused from the perforation process, and consequently VNMEA is able to sustain the physiological stability of cells during the long-term measurement. The optimization of physical conditions such as diameter, density, and height of nanowires can improve the viability and attachment of HEK293T cells to VNMEA and maintain long-term cell viability. (2) By virtue of the tight seal around the nanowires by the cellular membrane, the cytosolic composition in the cells is preserved without rupture of the membrane preventing homeostasis between the cell cytosol and the bath volume. (3) By applying optogenetic manipulation instead of traditional electrical or chemical manipulation, activated HEK293T cell potentials mediated by ChETA response instantly change the transmembrane voltage during an AP, resulting in a real-time negative feedback control. This self-control of the ChETA potential ensures long-term cell viability and recording duration.

## Discussion

Intracellular recording of the electrical activity of excitable cells provides rich information including subthreshold membrane potential changes as well as action potential generation. However, as intracellular recording methods inevitably promote cell damage during intracellular access, these methods are greatly limited in measuring long-term electrical events without signal run-down. Nanoscale electrodes built from one-dimensional nanomaterials such as nanowires and nanotubes have gained traction in electrophysiological studies of cellular activities because of their ability to be inserted into cells without cell damage by virtue of their small size relative to the cell size. Indeed, it has been reported that nanowire electrodes can be useful for intracellular electrical stimulation and signal detection of cells. For example, our previous studies demonstrated the long-term intracellular electrical stimulation response of PC 12 cells using vertically grown Si nanowires, and other studies have also shown signal detection of cells using nanowire electrodes with intracellular modes. However, despite their minimal invasiveness, long-term intracellular recording of cell electrical activities using nanowire electrodes has not yet been demonstrated. It has been verified that a vertical nanowire electrode array can record cell electrical activities for only about 80 min which is not enough to capture long-term cellular events taking place from several hours to days.

Considering that these reported studies have employed an external perforation force or depolarizing stimulus, in this study, we eliminated these two issues to support the extension of the measurement duration. Firstly, we designed a CVD-grown vertical nanowire multi electrode array (VNMEA) that can spontaneously internalize into the cell without an external perforation process. As CVD-grown Si nanowires have nanoscale diameters and high-aspect-ratios but are sufficiently rigid to be mechanically manipulated, the interiors of living cells are readily accessible by the nanowires. Moreover, by defining a pattern for the gold pads for the nanowires via lithography, we synthesized aligned arrays of vertical nanowires to facilitate electrical interfacing with individual single cells. This fabrication process is scalable enough to allow for a larger array that could realize simultaneous intracellular recording of a population of cells.

Furthermore, by exploiting optogenetic manipulation for activating cells instead of traditional excitation methods by electric fields or chemical agents, we were able to circumvent the drawbacks of these traditional stimulation methods and obtain long-term and high-quality intracellular and extracellular recordings of individual cells. Unlike traditional electrical stimulation, which has no built-in feedback, optogenetically-modified cells exhibited light-induced ion channel responses and self-regulated their transmembrane voltage during the generation of action potentials. This real-time negative feedback control consequently minimized cell damage from over-responsiveness. Possible photoelectric effects, which can be produced by the laser beam striking the tip of metal electrodes, are reduced by limiting the metal exposure to light by limiting the duration of light pulses and minimizing the total area of exposed metal surface. Although the electrode tip of VNMEA is coated with Pt film, we minimized the exposed metal tip with selective tip-etching and thus mitigated optical interferences. Unlike the artifacts of planar type electrodes (Pt film with same diameter pad), which can be as high as (40~80 mV), those of VNMEA are negligible (amplitude 5~10 mV) for recording cellular activities.

Applying two strategies, spontaneous internalization with CVD-grown nanowires and optogenetic manipulation, we recorded intracellular and extracellular electrical activities of optogenetically modified ChETA-expressing cells with optical stimulation. The electrochemical interface of nanowire electrodes at the intracellular and extracellular configurations form distinct equivalent circuits based on the components of the cell-electrode interface. We also successfully measured the ChETA potentials for 24 h with a 100% pacing rate and stable baseline maintained at 60 mV. These long-term physiological measurements of ChETA demonstrate the stable and compatible cell-coupling of VNMEA with optogenetic stimulation. The technique developed in this study which involves the combination of controllably grown nanowires with the selective excitation of a genetically targeted cell population has the potential for elucidating the long-term cellular dynamics of various cell types. Especially, for the point of view in neurons, various neuronal events can induce long-term electrophysiological changes in nervous system. For instance, neurons show diurnal variation in resting membrane potential. Also, timing-dependent activation of two interconnected neurons can mediate long-term potentiation (LTP) or long-term depression (LTD) of synapses. Membrane insertion of channels or newly innervated synapses can also affect electrophysiological properties of neurons. Without a proper device to investigate these time-consuming events, the mechanisms of these electrophysiological features are not yet fully understood. Long-term intracellular recordings in this study would contribute to revealing the cellular mechanism behind electrophysiological changes in cellular events. It would open up new possibilities of a range of studies and insights in biological and medical research.

## Methods

### Fabrication of VNMEA

The fabrication process of the VNMEA with conductive Pt layer for electrical recording is illustrated in Fig. 1. The fabrication of the device was reported in detail in our previous study^31,32^. Briefly, Si nanowires are epitaxially grown on the Si substrate by a vapor-liquid-solid (VLS) mechanism using chemical vapor deposition (CVD). The diameters, densities, and lengths of Si nanowires can be controlled by the growth parameters, for example, the size and concentration of Au catalyst particles and growth time, respectively. According to our previous studies, the optimum size of Si nanowires for intracellular use with cells was 3–5 μm in height and 60 nm in diameter^29,31^. A top-down CMOS device process was then used for electrode fabrication as follows (Fig. 1c–f). Double SiO_2_ passivation layers (each with a thickness of 300 nm) were used for electrode separation and for blocking the leakage of current from the substrate and culture media. The SiO_2_ passivation layers were deposited by a high density plasma CVD process for anisotropic deposition of the nanowire surface and substrate. Using a sputtering process, Pt film with a thickness of 100 nm was then deposited onto the surface of the Si nanowires to provide a conductive layer for recording of electrical signal (Fig. 1e). The second SiO_2_ passivation layer was deposited onto the substrate, and then the SiO_2_ layer of the nanowire electrodes was selectively etched out to expose the Pt of the nanowire surface by a buffered oxide etchant (BOE) (Fig. 1f). BOE used in the SiO_2_ etching process was a mixture of ammonium fluoride (NH_4_F), and hydrofluoric acid (HF) with a 6:1 volume ratio of 40% NH_4_F in water to 49% HF in water.

### HEK293T cells stably expressing ChETA

The method for generation of HEK293T cells stably expressing ChETA was previously described^34^. Breifly, ChETA-eYFP cDNA was amplified from pAAV-Ef1a-DIO-ChETA-EYFP (Addgene, Plasmid #26968) using forward (5′-GCTAGCGCCACCATGGACTATGG-3′) and reverse (5′-CTTAAGTTACTTGTACAGCTCGTCCATGC-3′) primers. The PCR product was then inserted into the restriction enzyme sites, NheI and AflII, of pcDNA5/FRT vector (Invitrogen, V601020). To generate cells that stably express ChETA-eYFP, Flp-HEK293T cells (Invitrogen, R75007) were co-transfected with ChETA-eYFP-containing pcDNA5/FRT and pOG44 vector and grown in Dulbeccos modified Eagles medium (DMEM) containing 10% FBS, penicillin (100 U/ml) and streptomycin (100 lg/ml). Using 100 mg/ml hygromycin, the transfected cells were selected 48 hours after transfection. The transfected cells were further confirmed by eYFP signal in experiments.

### Cell culture and Fixation

For whole-cell patch-clamp, nanowire-based recording or histology, cells were seeded and grown on VNMEA for 24 hours in DMEM. For cell viability tests, Calcein Red-Orange AM was incubated for 20 min in 36 °C medium and washed for 5 times with the Calcein-free medium. For histology, the cells were fixed using 4% paraformaldehyde (PFA). When the cytosol visualization is necessary, Alexa Fluor 555 hydrazide (Thermo Scientific, A20501MP) was intracellularly delivered after 30 min of 0.1% Tween-20 incubation for membrane permeabilization. The nuclei of HEK293T cells were stained by incubation in 4′,6-Diamidine-2′-phenylindole dihydrochloride (DAPI) solution. Confocal imaging was performed using LSM 700, Carl Zeiss. For electron microscopic imaging, cells were fixed with 2.5% glutaraldehyde for 1-day.

### Cell visualization and data acquisition

For recording of electrophysiological activity, live cells were visually identified using Olympus EX51WI (Olympus, Japan) microscope and ORCA-R2 camera (HAMAMATSU, Japan). Signals were obtained using Digitizer 1440 A and Multiclamp 700B (Molecular Devices, USA) using Clampex software (Molecular Devices, USA). The data were analyzed using Clampfit and Mini analysis software (Synaptosoft).

### Measurement of electrical activities by VNMEA

Of the 25 channels, 8 were selected – the maximum number of channels that the PCB system could record simultaneously. The typical features of a channel are the following: vertical population of >90% (vertical shape is defined by 90° ± 10°), height of 3~5 µm, and nanowire density of ~0.15 ea of nanowire/µm^2^.

Electrical activities were recorded under current clamp mode in recording buffer (in mM: 150 NaCl, 3 KCl, 10 HEPES, 2 MgCl_2_-6H_2_O, 2 CaCl_2_-2H_2_O, and 10 glucose) with pH adjusted to 7.35 with NaOH and osmolarity adjusted to 330 mOsm/L with sucrose. To activate ChETA, a 450 nm laser beam (MDL-III-450, CNI laser) was illuminated from the upper side of VNMEA. ChETA-induced electrical activities by illumination of laser beam were measured in current clamp mode. Typically, the cells were illuminated by laser of 5 Hz with a pulse width of 20 ms. For long-term recording, 1 Hz laser stimuli were illuminated with 20 ms durations.

### Whole-cell patch clamp measurement

All the whole-cell patch clamp was performed under the recording buffer (in mM: 150 NaCl, 3 KCl, 10 HEPES, 2 MgCl_2_-6H_2_O, 2 CaCl_2_-2H_2_O, and 10 glucose) with pH adjusted to 7.35 with NaOH. Patch electrodes (4–6 MΩ) were fabricated from standard-wall borosilicate glass (GC150F-10, Warner Instrument Corp., USA) using pipette puller. The pipettes were filled with internal solution containing K-gluconate, 125 mM; KCl, 10 mM; MgCl_2_, 1 mM; HEPES, 10 mM; EGTA, 0.02 mM; Mg-ATP, 4 mM; and Na2-GTP, 0.3 mM with pH adjusted to 7.35 and osmolality adjusted to 295 mOsmol/kg. For activation of ChETA, cells were illuminated by blue-light-laser of 5 Hz with a pulse width of 20 ms each.

## Supplementary information

Supplementary material.
