# Supplementary material for: Long-term Intracellular Recording of Optogenetically-induced Electrical Activities using Vertical Nanowire Multi Electrode Array
Source: Sci Rep. 2020 Mar 9;10:4279. doi: 10.1038/s41598-020-61325-3 (PMC7062878; doi:10.1038/s41598-020-61325-3)
Supplement: Supplementary file 1 — Supplementary material. [file 41598_2020_61325_MOESM1_ESM.docx]

Supporting Information for

Long-term Intracellular Recording of Optogenetically-induced Electrical Activities using Vertical Nanowire Multi Electrode Array

Jisoo Yoo^1†^, Hankyul Kwak^2†^, Juyoung Kwon^1†^, Go Eun Ha^2^, Elliot H. Lee^2^, Seungwoo Song^3^, Jukwan Na^1^, Hyo-Jung Lee^1^, Jaejun Lee^1^, Areum Hwangbo^2^, Eunkyung Cha^1^, Youngcheol Chae^3^, Eunji Cheong^2,4*^ and Heon-Jin Choi^1*^

^1^Department of Materials Science and Engineering, Yonsei University, Seoul 03722, Republic of Korea.

^2^Department of Biotechnology, College of Life Science and Biotechnology, Yonsei University, Seoul 03722, Republic of Korea.

^3^Department of Electrical and Electronic Engineering, Yonsei University, Seoul 03722, Republic of Korea.

^4^Center for Nanomedicine, Institute for Basic Science (IBS), Seoul 03722, Republic of Korea.

†These authors contributed equally to this work.

*Correspondence to: eunjicheong@yonsei.ac.kr, [hjc@yonsei.ac.kr](mailto:hjc@yonsei.ac.kr)

**Scanning electron microscope (SEM) and Focused Ion Beam (FIB) milling analysis.**

We fixed the cultured HEK293T cells on VNMEA device and cross-sectioned the cell by a high-resolution Cross Beam Focused Ion Beam – Field Effect SEM (FE-SEM) and observed by Scanning Electron Microscope (SEM).

Cells on the VNMEA device were firstly fixed with 2% glutaraldehyde – paraformaldehyde in 0.1M phosphate buffer (PB), pH 7.4 for 6 hours and washed two times for 30 minutes in 0.1M PB. They were post-fixed with 1% osmium tetroxide (OsO_4_) dissolved in distilled water at room temperature for 1.5 hours in a dark condition, and then samples were rinsed three times with 0.1 M phosphate buffer for 10 minutes each. The samples were dehydrated in ascending gradual series (30, 50, 70, 80, 90 and 95%) of ethanol at room temperature for 15 minutes each. The last dehydration step was in 100% ethanol with two changes within 20 minutes at room temperature. Finally, the cells were subjected to critical point dryer (LEICA EM CPD300, Austria) and coated with a 5 nm-thickness of platinum (Pt) film by Ion Coater (LEICA EM ACE600, Austria) After the cell fixation, samples were examined and photographed with a Scanning Electron Microscopy (FE-SEM; Merin, Carl ZEISS, Germany) 2Kv. The Cells were cross-sectioned from the substrate by a high-resolution Cross Beam FIB-FESEM (Helios Nanolab 600).


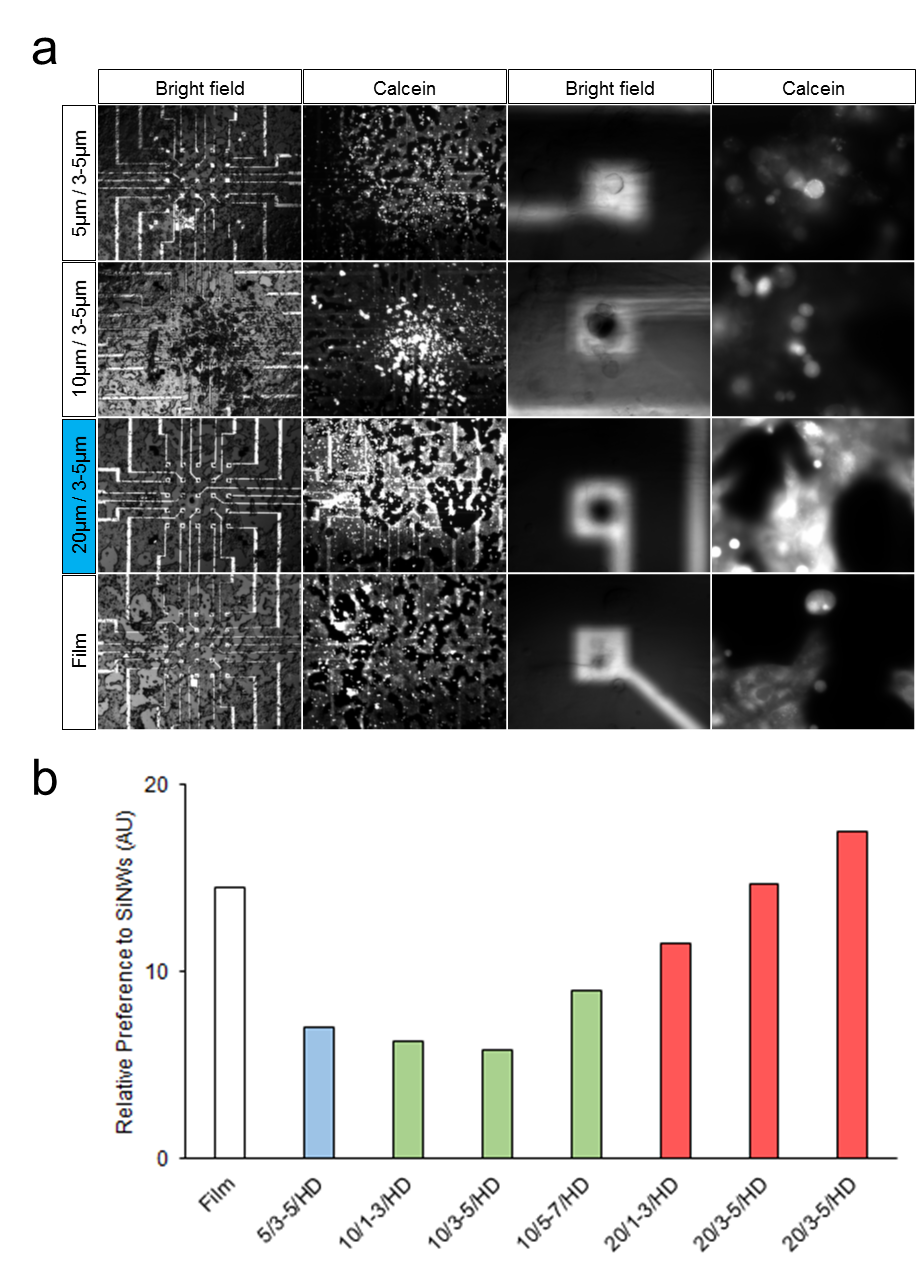


**Figure S1. Biocompatibility test of VNMEA with various SiNW dot sizes and heights. a.** Calcein incubated HEK293T cells on VNMEAs. Calcein signals persisting in cells show viable cells. **b**. A bar-graph of the relative preference of HEK293T cells to SiNWs. 20µm-dot and 3-5µm-height SiNWs show comparable values with HEK293T preference for film.


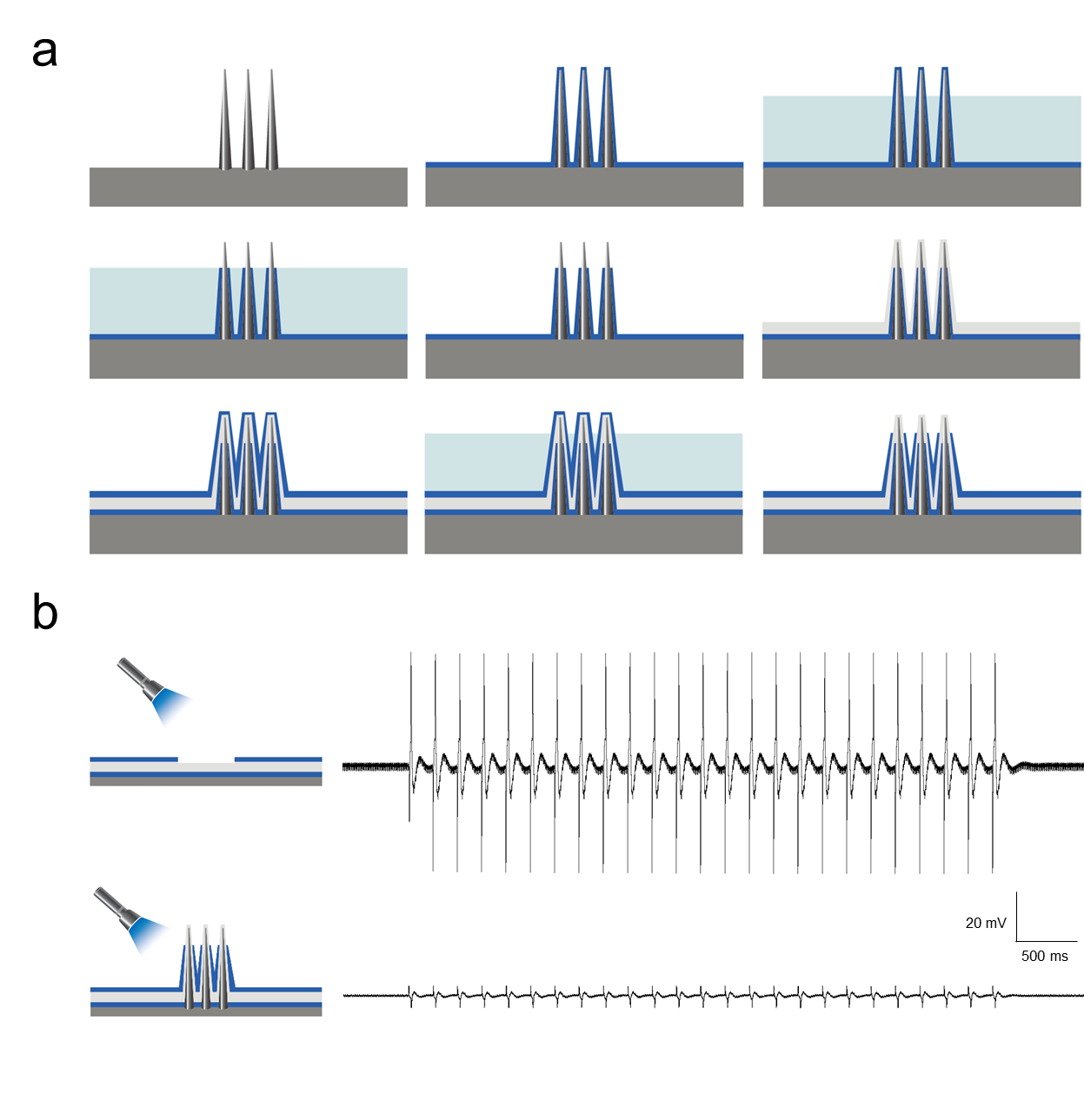


**Figure S2. Experimental solutions for minimizing light-induced artifacts. a.** Fabrication process of nano-electrode tip etching which minimizes metal (Pt; light grey) exposure to mitigate light-induced artifacts as well to make the leak resistance (Rs) very high to prevent current leakage. **b.** Light-induced artifact (artificial photocurrent) depending on the geometry of electrodes. The amplitudes of artifacts generated by planar type electrodes are particularly higher (40 ~ 80 mV) than those of nanowire type electrodes (5 ~ 10 mV), due to the large surface area of planar electrodes, thus interfering with neural recordings.


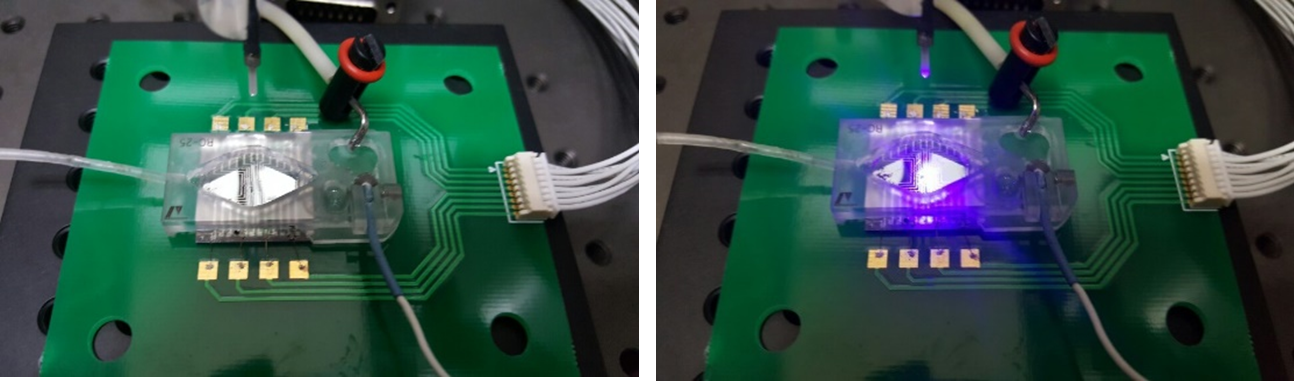


**Figure S3. An intracellular electrical recording system using VNMEA with a PCB system. a.** Digital camera image of PCB consisting of 8 channels with VNMEA, before the stimuli delivered by blue diode laser. **b.** Digital camera image of PCB with VNMEA, during stimuli delivery by a blue diode laser.
